# Viral infections in etiology of mental disorders: a broad analysis of cytokine profile similarities – a narrative review

**DOI:** 10.3389/fcimb.2024.1423739

**Published:** 2024-08-14

**Authors:** Piotr Lorkiewicz, Napoleon Waszkiewicz

**Affiliations:** Department of Psychiatry, Medical University of Bialystok, Białystok, Poland

**Keywords:** SARS-CoV-2, influenza, HIV, virus, interleukin, inflammation, schizophrenia, depression

## Abstract

The recent pandemic caused by the SARS-CoV-2 virus and the associated mental health complications have renewed scholarly interest in the relationship between viral infections and the development of mental illnesses, a topic that was extensively discussed in the previous century in the context of other viruses, such as influenza. The most probable and analyzable mechanism through which viruses influence the onset of mental illnesses is the inflammation they provoke. Both infections and mental illnesses share a common characteristic: an imbalance in inflammatory factors. In this study, we sought to analyze and compare cytokine profiles in individuals infected with viruses and those suffering from mental illnesses. The objective was to determine whether specific viral diseases can increase the risk of specific mental disorders and whether this risk can be predicted based on the cytokine profile of the viral disease. To this end, we reviewed existing literature, constructed cytokine profiles for various mental and viral diseases, and conducted comparative analyses. The collected data indicate that the risk of developing a specific mental illness cannot be determined solely based on cytokine profiles. However, it was observed that the combination of IL-8 and IL-10 is frequently associated with psychotic symptoms. Therefore, to assess the risk of mental disorders in infected patients, it is imperative to consider the type of virus, the mental complications commonly associated with it, the predominant cytokines to evaluate the risk of psychotic symptoms, and additional patient-specific risk factors.

## 1 Introduction

In the last three years, the SARS-CoV-2 pandemic has prompted numerous studies investigating the neuropsychiatric and systemic consequences of infection with this virus. Evidence indicates that the risk of developing anxiety disorders (AD), depression, or substance abuse disorders is higher in patients after COVID-19 compared to those hospitalized for other infections or reasons (Xie et al., 2022). The emergence of such an aggressive pathogen and its associated complications, including mental health issues, has revitalized discussions on the etiology of mental disorders, providing an opportunity to refine existing knowledge. However, SARS-CoV-2 is not the only virus that can potentially influence the development of neuropsychiatric disorders, and this concept is relatively old. Emil Kraepelin advocated the theory of autoinfection and focal infections as etiological factors for dementia praecox, now called schizophrenia (SCZ) (Noll, 2004). In 1893, after the Asian flu pandemic, cases of post-influenza delirium were documented, distinguished from febrile delirium by their persistence after the fever subsided. Some cases also exhibited ‘delirium’ prior to the rise in temperature. Additionally, psychosis associated with influenza was noted to be significantly more frequent compared to other febrile illnesses of similar severity (Althaus, 1893). After the Spanish flu epidemic, Karl Menninger described numerous cases of ‘dementia praecox’ occurring during acute infection. Some patients experienced symptom resolution, while others exhibited symptoms persisting for up to five years post-infection (Influenza and schizophrenia, 1994). Menninger’s theory of ‘reversible dementia praecox’ proposed that dementia praecox was a somatopsychosis—a mental manifestation of encephalitis, with reversibility dependent on the inflammation’s duration and severity (Menninger, 1922). Contrastingly, some later studies negated the association between viral infections and mental disorders. Mid-20th century research into post-influenza depression and the correlation between other mental illnesses and antibody titers against various viruses found no significant relationships (Pokorny et al., 1973; Sinanan and Hillary, 1981; King et al., 1985). Modern researchers investigating the impact of infections on the development of mental disorders consider potential etiopathogenetic factors including influenza, HSV-1, HSV-2, EBV, CMV, measles, rubella (RV), mumps, polio, Coxsackie virus B4, HIV, BoDV, T. gondii, and Treponema pallidum (Yolken and Torrey, 2008). Questions have also arisen regarding whether, in considering the impact of viruses on mental illness development, we should exclude viruses with relatively recent human infection histories, such as HIV, or those with declining infection rates, such as RV, mumps, and measles. The issue of seasonality in mental illnesses also warrants consideration, necessitating scrutiny of viruses exhibiting seasonal patterns, such as influenza. However, a recent meta-analysis did not substantiate the proposed influence of birth season on the risk of developing BD or SCZ, as hypothesized 25 years ago (Torrey et al., 1997; Saleh et al., 2021). Additionally, urban birth and upbringing have been shown to increase SCZ risk, which some authors suggest may be related to accelerated and facilitated pathogen transmission due to high population density and a large number of interpersonal contacts (Eaton et al., 2000; Pedersen and Mortensen, 2001; Harrison et al., 2003). Exploration of viral impacts on mental illnesses is grounded in immunology, pathophysiology, and biochemistry. Numerous mechanisms exist through which viruses could influence the brain and mental disorder development. Viruses may enter the body via various routes, establish systemic or localized infections, penetrate the brain via the bloodstream or neuronal routes, and traverse or breach the blood-brain barrier. They may cause acute, persistent, or latent infections—potentially lifelong and reactivating. Viral replication can be cytopathic, destroying CNS cells, or non-cytopathic (Koyuncu et al., 2013; Nath and Johnson, 2022). Direct viral impacts on the brain include cellular damage during replication or interaction of viral proteins with brain cell receptors, while indirect impacts involve cytokine and chemokine induction, neurotransmission alterations, increased excitotoxicity, and oxidative stress (Chhatbar and Prinz, 2021; Rocamonde et al., 2023). Viral infections within the CNS elicit robust immune responses, paralleling those observed in mental illnesses. Given the multitude of mechanisms by which viruses can affect the brain, and considering the high frequency of infections and the diversity of viruses, including those yet undiscovered, it is imperative to contemplate viral impacts on mental disorder development. The SARS-CoV-2 pandemic has underscored this relevance, reigniting discussions on virus-mental illness connections. Despite the pandemic is in retreat, its mental health repercussions may persist, particularly in generations born or in early development during its peak. Thus, it is crucial to elucidate whether viruses contribute to mental illness, and if so, whether the risk is influenced solely by systemic responses or also by the type of virus and its mechanism of action.

### 1.1 Objectives of the review

This work aims to enhance our understanding of the intersection between viral infections and mental health, ultimately aiding in better clinical assessments and preparedness for future epidemics as well as new or mutated viruses. We also aim to expand and enrich the existing knowledge concerning the etiology of mental disorders and to identify possible future research directions. This goal encompasses:

• presenting the inflammatory basis of mental disorders and current knowledge on cytokine disturbances in mental illnesses

• creating cytokine profiles of mental disorders

• concisely and critically presenting the current knowledge on the connections between specific, common viral infections and the occurrence of mental disorders

• creating cytokine profiles of viral infections and comparing them to the cytokine profiles of mental disorders

• identifying other possible mechanisms of viral impact on the CNS and other factors increasing the risk of developing mental disorders

• critically discussing the gathered information, interpreting it, and drawing conclusions from it

### 1.2 Methods of this review article

Since this work is a narrative review, we did not establish strict inclusion criteria for the articles. We selected articles that met just several key criteria: they were meta-analyses, original research papers, or large systematic reviews, and their topics were related to mental disorders associated with viral diseases (or negated such an association), cytokine disturbances in mental disorders and viral diseases, and other mechanisms of viral impact on the CNS. Additionally, we included relevant supporting literature. Exclusion criteria included articles not available in English due to concerns about errors from incorrect translation and incomplete understanding of the text, as well as to ensure that most readers could read the source literature in its original form if desired. We also excluded studies focusing on the occurrence of viral diseases in populations with already diagnosed mental disorders, as this data was not directly relevant to assessing the first-time occurrence of a mental illness and its association with prior viral infection. A literature search was conducted in PubMed, Scopus, and Google Scholar databases. The search strategy consisted of the following keywords: ‘cytokine’, ‘inflammation’, ‘biomarkers’, ‘psychiatric sequel’, ‘mental disorder’, ‘psychosis’, ‘mental health’, ‘COVID-19’. ‘SARS-CoV-2’, ‘Influenza’, ‘HIV’, ‘HSV’, ‘CMV’, ‘EBV’, ‘BoDV’, ‘ Rubella’ as well as variations and combinations of these terms. The strategy for creating cytokine profiles is described later in the work, in the footnotes to the relevant tables.

## 2 Inflammation

To elucidate the role of viruses in the etiopathogenesis of mental disorders, it is essential to explain the rationale behind this hypothesis. The proposed mechanism by which viruses could induce psychiatric disorders is multifaceted, with inflammation being the most well-studied and likely pathway. Extensive research has demonstrated the impact of inflammation on major depressive disorder (MDD) (Gałecki and Talarowska, 2018; Lee and Giuliani, 2019; Beurel et al., 2020; Roohi et al., 2021), BD (Brietzke et al., 2009; Berk et al., 2011; Modabbernia et al., 2013), SCZ (Müller et al., 2015; Müller, 2018; Mongan et al., 2020), and to a lesser extent, obsessive-compulsive disorder (OCD) (Rodríguez et al., 2017; Cosco et al., 2019; Westwell-Roper et al., 2022). Notably, in SCZ, there is considerable discussion regarding the effects of pre- or perinatal exposure to infections or generalized inflammation on immune reactivity throughout life, thereby increasing the risk of developing SCZ (Brown, 2006; Brown, 2011; Müller, 2018). Inflammation involves the production of pro- and anti-inflammatory cytokines. Proinflammatory cytokines, such as interleukin-1β (IL-1β), interleukin-2 (IL-2), interleukin-6 (IL-6), tumor necrosis factor α (TNF-α), and interferon gamma (IFN-γ), are primarily produced by Th1 lymphocytes and M1 macrophages, as well as microglial cells within the central nervous system (CNS). Conversely, anti-inflammatory cytokines, including interleukin-4 (IL-4), interleukin-5 (IL-5), interleukin-10 (IL-10), interleukin-13 (IL-13), and transforming growth factor β (TGF-β), are secreted by Th2 lymphocytes, M2 macrophages, and astroglial cells in the CNS (Berger, 2000; Raphael et al., 2015). Th1 cells are crucial in the immune response to intracellular bacteria and viruses, with M1 macrophages activating lymphocytes at inflammation sites. Th2 cells are primarily involved in humoral and allergic responses, while M2 macrophages facilitate wound healing, tissue repair, and remodeling post-inflammation (Romagnani, 1999; Raphael et al., 2015). Pro-inflammatory cytokines render the brain more susceptible to stress and disrupt its functions by adversely affecting neuroplasticity, hippocampal neurogenesis, synaptogenesis, and synaptic plasticity. These cytokines also induce cellular apoptosis, promote free radical production, and act as neuromodulators, disrupting neuroendocrine and neurochemical pathways (Fuchs et al., 2004; Khairova et al., 2009; Cattaneo et al., 2015). Although cytokines can be produced and released within the CNS itself (Ja S et al., 2012; Choi et al., 2014; Marisa et al., 2018), peripheral cytokines also reach and influence the CNS, increasing blood-brain barrier (BBB) permeability, affecting astrocytes and microglia, which in turn produce more cytokines, perpetuating CNS inflammation (Miller et al., 2009; Miller et al., 2013; Cheng et al., 2018; Sun et al., 2022). The condition characterized by the most severe cytokine disturbances is a cytokine storm, a systemic pathological immune response in which a positive feedback loop occurs between cytokines and immune cells, leading to an uncontrolled elevation of cytokine levels. A peripheral cytokine storm does not necessarily correlate with a similar phenomenon in the brain. During systemic inflammation, increased BBB permeability allows more cytokines to enter the CNS. If the brain’s anti-inflammatory systems promptly balance this influx, an inflammatory cascade and cytokine storm may be averted. Cytokine abnormalities in the brain can lead to various forms of neuroinflammation. Acute and severe neuroinflammation manifests as encephalitis, but milder, chronic neuroinflammation can occur, termed mild neuroinflammation (MNI) or mild encephalitis (ME). These milder forms may primarily cause psychiatric symptoms, with neurological symptoms emerging as the condition progresses. Another complication is inflammatory encephalopathy, characterized by lasting brain dysfunction due to severe systemic inflammation, without direct brain inflammation. Extreme cytokine disorders, such as cytokine storms within the brain, typically lead to severe neuroinflammation or encephalitis, although this is not always the case (Bechter, 2019; Tetsuka et al., 2021; Vengalil et al., 2023).

The complexity of biochemical alterations during inflammation poses significant challenges in identifying specific patterns that increase the risk of mental disorders. Consequently, this study concentrates on cytokine changes, which can be systematically analyzed. Each cytokine exerts multidirectional effects and interacts with other cytokines, inflammatory factors, chemokines, and oxidative stress factors, resulting in a specific cytokine “mixture” that can differentially impact the central nervous system (CNS). This has led to efforts to establish cytokine profiles for specific mental disorders. **Table 1** provides an overview of the direction of cytokine and chemokine concentration changes in patients with specific mental disorders, based on a review of the literature. It is noticeable that these changes are not consistent in every case. These differences may be due to factors such as disease progression, comorbidities, and treatment methods. However, in all cases, it is evident that cytokine disturbances are present to a greater or lesser extent, and that there is most often an increase in the concentration of pro-inflammatory cytokines—particularly IL-6 and TNF-alpha. This finding, though nonspecific, supports the inflammatory theory of mental disorders. Despite the fact that the cytokine disturbances described in the literature are not entirely consistent with each other, it is possible to identify which cytokines most frequently undergo changes in their concentration and the direction of these changes, i.e., increase or decrease. The changes determined in this way create a ‘cytokine profile’ that occurs in a given disorder. In our work, we have attempted to create such profiles, and the results are illustrated in **Table 2**. Constructing these profiles is crucial and holds potential future value, as understanding the physical diseases or infections that elicit cytokine secretion patterns akin to those observed in mental disorders may facilitate the prediction of psychiatric disorder development in patients.

**Table 1:** Cytokine changes in mental disorders.

Cytokine	MDD	BD	SCZ	OCD	PTSD	ASD	SUICIDE	FEP
IL-1α	**NC** (Cassano et al., 2017)					**NC** (Masi et al., 2015; Saghazadeh et al., 2019a; Yuan et al., 2019)		
IL-1β	**NC** (Dowlati et al., 2010; Haapakoski et al., 2015; Cassano et al., 2017; Köhler et al., 2017; Yuan et al., 2019) **↑** (Chang and Yung Chieh, 2010)	**NC** (Munkholm et al., 2013a; Barbosa et al., 2014; Yuan et al., 2019) **↑** (Najjar et al., 2013)	**NC** (Yuan et al., 2019) **↑** (Miller et al., 2011; Lesh et al., 2018; Momtazmanesh et al., 2019; Halstead et al., 2023)	**↓** (Gray and Bloch, 2012; Najjar et al., 2013) **↑** (Karagüzel et al., 2019) **NC** (Rodríguez et al., 2017)	**↑** (Passos et al., 2015; Yuan et al., 2019; Sun et al., 2021) **NC** (Imai et al., 2018; Yang and Jiang, 2020)	**↑** (Masi et al., 2015; Saghazadeh et al., 2019a; Yuan et al., 2019; Zhao et al., 2021) **NC** (Eftekharian et al., 2018)	**↑** (Black and Miller, 2015; Serafini et al., 2020)	**NC** (Dunleavy et al., 2022) **↑** (Upthegrove et al., 2014; Corsi Zuelli et al., 2019)
IL-2	**NC** (Dowlati et al., 2010; Cassano et al., 2017; Köhler et al., 2017; Yuan et al., 2019) **↓** (Chang and Yung Chieh, 2010; Najjar et al., 2013)	**NC** (Chang and Yung Chieh, 2010; Modabbernia et al., 2013; Munkholm et al., 2013a; Barbosa et al., 2014; Rowland et al., 2018; Yuan et al., 2019) **↑** (Najjar et al., 2013)	**NC** (Momtazmanesh et al., 2019; Yuan et al., 2019) **↑** (Chang and Yung Chieh, 2010; Lesh et al., 2018; Halstead et al., 2023) **↓** (Potvin et al., 2008)	**↑** (Şimşek et al., 2016)	**NC** (Yuan et al., 2019; Yang and Jiang, 2020)	**↓** (Eftekharian et al., 2018) **NC** (Saghazadeh et al., 2019a)	**↓** (Black and Miller, 2015; Ducasse et al., 2015; Yuan et al., 2019; Serafini et al., 2020)	**↑** (Petrikis et al., 2015) **NC** (Noto et al., 2014; Upthegrove et al., 2014; Dunleavy et al., 2022)
IL-4	**NC** (Dowlati et al., 2010; Cassano et al., 2017; Köhler et al., 2017; Yuan et al., 2019)	**↑** (Chang and Yung Chieh, 2010; Modabbernia et al., 2013; Munkholm et al., 2013a; Najjar et al., 2013; Barbosa et al., 2014; Yuan et al., 2019) **NC** (Rowland et al., 2018)	**NC** (Momtazmanesh et al., 2019; Yuan et al., 2019) **↓** (Halstead et al., 2023)	**NC** (Şimşek et al., 2016)	**NC** (Passos et al., 2015; Yuan et al., 2019) **↑** (Sun et al., 2021) **↓** (Sun et al., 2021)	**NC** (Masi et al., 2015; Eftekharian et al., 2018; Saghazadeh et al., 2019b; Yuan et al., 2019; Zhao et al., 2021) **↑** (Zhao et al., 2021)	**↓** (Ducasse et al., 2015; Yuan et al., 2019; Serafini et al., 2020)	**NC** (Noto et al., 2014; Upthegrove et al., 2014; Corsi Zuelli et al., 2019; Dunleavy et al., 2022)
IL-5	**NC** (Cassano et al., 2017; Köhler et al., 2017; Yuan et al., 2019)	**NC** (Munkholm et al., 2013a; Barbosa et al., 2014; Yuan et al., 2019)				**↑** (Saghazadeh et al., 2019b)		
IL-6	**↑** (Chang and Yung Chieh, 2010; Dowlati et al., 2010; Fontenelle et al., 2012; Najjar et al., 2013; Goldsmith et al., 2016; Köhler et al., 2017; Yuan et al., 2019; Osimo et al., 2020) **NC** (Cassano et al., 2017; Renner et al., 2022)	**NC** (Kunz et al., 2011; Barbosa et al., 2014; Rowland et al., 2018; Yuan et al., 2019) **↑** (Chang and Yung Chieh, 2010; Modabbernia et al., 2013; Najjar et al., 2013; Barbosa et al., 2014; Goldsmith et al., 2016; Rowland et al., 2018)	**↑** (Potvin et al., 2008; Chang and Yung Chieh, 2010; Kunz et al., 2011; Miller et al., 2011; Goldsmith et al., 2016; Lesh et al., 2018; Momtazmanesh et al., 2019; Yuan et al., 2019; Halstead et al., 2023)	**↑** (Najjar et al., 2013; Karagüzel et al., 2019) **NC** (Gray and Bloch, 2012; Najjar et al., 2013; Şimşek et al., 2016; Rodríguez et al., 2017)	**↑** (Passos et al., 2015; de Oliveira et al., 2018; Imai et al., 2018; Yuan et al., 2019; Sun et al., 2021) **NC** (Yang and Jiang, 2020; Renner et al., 2022)	**↑** (Masi et al., 2015; Eftekharian et al., 2018; Saghazadeh et al., 2019a; Yuan et al., 2019; Zhao et al., 2021)	**↑** (Black and Miller, 2015; Yuan et al., 2019; Serafini et al., 2020) **↓** (Yuan et al., 2019)	**↑** (Kubistova et al., 2012; Noto et al., 2014; Upthegrove et al., 2014; Petrikis et al., 2015; Corsi Zuelli et al., 2019; Pillinger et al., 2019; Dunleavy et al., 2022)
IL-8	**↓** (Yuan et al., 2019) **↑** (Yuan et al., 2019) **NC** (Dowlati et al., 2010; Cassano et al., 2017; Köhler et al., 2017)	**NC** (Modabbernia et al., 2013; Munkholm et al., 2013a; Barbosa et al., 2014; Yuan et al., 2019) **↑** (Chang and Yung Chieh, 2010; Misiak et al., 2020)	**↑** (Chang and Yung Chieh, 2010; Momtazmanesh et al., 2019; Halstead et al., 2023)	**NC** (Rodríguez et al., 2017) **↑** (Fontenelle et al., 2012)	**NC** (Yuan et al., 2019)	**↑** (Masi et al., 2015; Yuan et al., 2019; Zhao et al., 2021) **NC** (Eftekharian et al., 2018; Saghazadeh et al., 2019a)	**↓** (Black and Miller, 2015)*	**NC** (Kubistova et al., 2012; Dunleavy et al., 2022)
IL-10	**↓** (Yuan et al., 2019) **NC** (Chang and Yung Chieh, 2010; Dowlati et al., 2010; Cassano et al., 2017; Renner et al., 2022) **↑** (Köhler et al., 2017; Yuan et al., 2019)	**↑** (Chang and Yung Chieh, 2010; Kunz et al., 2011; Modabbernia et al., 2013; Lesh et al., 2018) **NC** (Munkholm et al., 2013a; Rowland et al., 2018)	**NC** (Yuan et al., 2019) **↑** (Chang and Yung Chieh, 2010; Kunz et al., 2011; Momtazmanesh et al., 2019; Halstead et al., 2023) **↓** (Momtazmanesh et al., 2019)	**NC** (Şimşek et al., 2016)	**NC** (Passos et al., 2015; Yuan et al., 2019) **↑** (de Oliveira et al., 2018; Renner et al., 2022) **↓** (Sun et al., 2021)	**NC** (Masi et al., 2015; Yuan et al., 2019) **↓** (Saghazadeh et al., 2019b)	**↑** (Yuan et al., 2019)	**NC** (Kubistova et al., 2012; Petrikis et al., 2015; Dunleavy et al., 2022) **↑** (Noto et al., 2014; Corsi Zuelli et al., 2019)
IL-12	**↑** (Köhler et al., 2017; Yuan et al., 2019; Osimo et al., 2020) **NC** (Cassano et al., 2017)	**NC** (Munkholm et al., 2013a; Barbosa et al., 2014)	**↑** (Miller et al., 2011; Momtazmanesh et al., 2019; Sun et al., 2021) **↓** (Halstead et al., 2023)			**NC** (Masi et al., 2015; Saghazadeh et al., 2019a)		**↑** (Dunleavy et al., 2022)
IL-13	**↑** (Köhler et al., 2017; Yuan et al., 2019) **NC** (Cassano et al., 2017)					**NC** (Saghazadeh et al., 2019b)		
IL-17	**NC** (Köhler et al., 2017; Yuan et al., 2019)		**NC** (Momtazmanesh et al., 2019)	**↑** (Şimşek et al., 2016)	**↑** (Sun et al., 2021)	**NC** (Masi et al., 2015; Saghazadeh et al., 2019a; Yuan et al., 2019; Zhao et al., 2021) **↑** (Eftekharian et al., 2018; Zhao et al., 2021)		**NC** (Noto et al., 2014; Petrikis et al., 2015; Fang et al., 2018; Saghazadeh et al., 2019a) **↑** (Pillinger et al., 2019; Dunleavy et al., 2022)
IL-18	**↑** (Köhler et al., 2017; Yuan et al., 2019; Osimo et al., 2020)					NC (Saghazadeh et al., 2019a)		
TNF-α	**↑** (Dowlati et al., 2010; Najjar et al., 2013; Goldsmith et al., 2016; Köhler et al., 2017; Yuan et al., 2019; Osimo et al., 2020) **↓** (Yuan et al., 2019) **NC** (Haapakoski et al., 2015; Cassano et al., 2017)	**↑** (Modabbernia et al., 2013; Munkholm et al., 2013a; Munkholm et al., 2013b; Barbosa et al., 2014; Goldsmith et al., 2016; Rowland et al., 2018; Yuan et al., 2019) **NC** (Kunz et al., 2011)	**NC** (Kunz et al., 2011; Yuan et al., 2019) **↑** (Miller et al., 2011; Goldsmith et al., 2016; Momtazmanesh et al., 2019; Halstead et al., 2023)	**↑** (Najjar et al., 2013; Şimşek et al., 2016; Karagüzel et al., 2019) **↓** (Najjar et al., 2013) NC (Gray and Bloch, 2012; Rodríguez et al., 2017)	**NC** (Imai et al., 2018; Yuan et al., 2019) **↑** (Passos et al., 2015; Yang and Jiang, 2020; Sun et al., 2021)	**NC** (Yuan et al., 2019; Zhao et al., 2021) **↑** (Guloksuz et al., 2017; Eftekharian et al., 2018; Saghazadeh et al., 2019a; Zhao et al., 2021)	**NC** (Yuan et al., 2019) **↑** (Serafini et al., 2020)	**NC** (Dunleavy et al., 2022) **↑** (Kubistova et al., 2012; Noto et al., 2014; Upthegrove et al., 2014; Corsi Zuelli et al., 2019; Pillinger et al., 2019)
IFN-γ	**↑** (Yuan et al., 2019) **↓** (Köhler et al., 2017; Yuan et al., 2019) **NC** (Dowlati et al., 2010; Cassano et al., 2017)	**NC** (Modabbernia et al., 2013; Munkholm et al., 2013a; Barbosa et al., 2014; Rowland et al., 2018; Yuan et al., 2019)	**NC** (Yuan et al., 2019) **↑** (Miller et al., 2011; Lesh et al., 2018; Momtazmanesh et al., 2019; Halstead et al., 2023) **↓** (Momtazmanesh et al., 2019; Halstead et al., 2023)		**↑** (Passos et al., 2015; Yuan et al., 2019; Yang and Jiang, 2020; Sun et al., 2021)	**↑** (Masi et al., 2015; Saghazadeh et al., 2019a; Yuan et al., 2019) **NC** (Eftekharian et al., 2018)	**NC** (Yuan et al., 2019) **↓** (Serafini et al., 2020)	**↑** (Pillinger et al., 2019; Dunleavy et al., 2022) **NC** (Noto et al., 2014; Upthegrove et al., 2014; Corsi Zuelli et al., 2019)
TGF-β	**NC** (Köhler et al., 2017; Yuan et al., 2019)	**NC** (Munkholm et al., 2013a; Barbosa et al., 2014)	**↑** (Miller et al., 2011; Momtazmanesh et al., 2019)			**↓** (Masi et al., 2015) **NC** (Eftekharian et al., 2018; Saghazadeh et al., 2019b)	**↑** (Ducasse et al., 2015; Yuan et al., 2019; Serafini et al., 2020)	**NC** (Petrikis et al., 2015) **↑** (Corsi Zuelli et al., 2019; Pillinger et al., 2019)
CRP	**↑** (Cassano et al., 2017; Yuan et al., 2019; Osimo et al., 2020)	**↑** (Rowland et al., 2018)	**↑** (Yuan et al., 2019; Halstead et al., 2023)		**NC** (Passos et al., 2015; Imai et al., 2018; Yuan et al., 2019) **↑** (Yang and Jiang, 2020)		**↑** (Yuan et al., 2019)	
sTNF-R1		**↑** (Hope et al., 2009; Modabbernia et al., 2013; Munkholm et al., 2013a; Munkholm et al., 2013b; Barbosa et al., 2014; Rowland et al., 2018; Yuan et al., 2019)	**↑** (Potvin et al., 2008)	**↑** (Fontenelle et al., 2012)		**NC** (Saghazadeh et al., 2019a)		
sTNF-R2	**↑** (Köhler et al., 2017; Yuan et al., 2019)	**NC** (Munkholm et al., 2013a; Barbosa et al., 2014; Yuan et al., 2019)		**↑** (Fontenelle et al., 2012)		**NC** (Saghazadeh et al., 2019a)		
IL-1RA	**↑** (Goldsmith et al., 2016; Köhler et al., 2017; Yuan et al., 2019)	**NC** (Munkholm et al., 2013a; Barbosa et al., 2014; Yuan et al., 2019) **↑** (Modabbernia et al., 2013; Najjar et al., 2013; Goldsmith et al., 2016; Rowland et al., 2018)	**↑** (Potvin et al., 2008; Goldsmith et al., 2016; Yuan et al., 2019; Halstead et al., 2023)		**↑** (Toft et al., 2018)	**NC** (Masi et al., 2015; Yuan et al., 2019) **↓** (Saghazadeh et al., 2019b)		
sIL-6R	**NC** (Köhler et al., 2017; Yuan et al., 2019)	**↑** (Modabbernia et al., 2013; Munkholm et al., 2013a; Rowland et al., 2018; Yuan et al., 2019)	**NC** (Yuan et al., 2019)		**NC** (Imai et al., 2018; Yuan et al., 2019)			
sIL-2R	**↑** (Najjar et al., 2013; Goldsmith et al., 2016; Köhler et al., 2017; Yuan et al., 2019; Osimo et al., 2020)	**↑** (Modabbernia et al., 2013; Munkholm et al., 2013a; Munkholm et al., 2013b; Barbosa et al., 2014; Goldsmith et al., 2016; Rowland et al., 2018; Yuan et al., 2019)	**↑** (Potvin et al., 2008; Miller et al., 2011; Goldsmith et al., 2016; Yuan et al., 2019; Halstead et al., 2023)		**NC** (Yuan et al., 2019)	**NC** (Saghazadeh et al., 2019a)		**↑** (Upthegrove et al., 2014)
CCL2	**↑** (Köhler et al., 2017; Yuan et al., 2019) NC (Cassano et al., 2017)	**NC** (Modabbernia et al., 2013; Yuan et al., 2019) **↑** (Misiak et al., 2020)				**↑** (Masi et al., 2015; Yuan et al., 2019; Zhao et al., 2021)		
CCL11	**↑** (Yuan et al., 2019) **NC** (Cassano et al., 2017)	**↑** (Barbosa et al., 2014; Misiak et al., 2020)				**↑** (Masi et al., 2015; Yuan et al., 2019; Zhao et al., 2021)		
CXCL4	**↑** (Yuan et al., 2019)							
CCL5					**↑** (Pan et al., 2021)	**NC** (Masi et al., 2015)		
CCL4					**↑** (Pan et al., 2021)			
CXCL7	**↑** (Yuan et al., 2019)							
CCL3/ MIP-1α					**↑** (Pan et al., 2021)	**NC** (Masi et al., 2015)		
CXCL10	**NC** (Cassano et al., 2017)	**↑** (Barbosa et al., 2014; Misiak et al., 2020)						
NT4/5		**↑** (Yuan et al., 2019)						
NT3		**↑** (Yuan et al., 2019)						
NGF	**↓** (Yuan et al., 2019)		**↓** (Yuan et al., 2019)			**↑** (Zhao et al., 2021)		
VEGF	**↑** (Yuan et al., 2019) **NC** (Cassano et al., 2017)						**↑** (Serafini et al., 2020)	
IGF-1	**↑** (Yuan et al., 2019)	**↑** (Yuan et al., 2019)						
S100			**↑** (Yuan et al., 2019)			**↑** (Guloksuz et al., 2017)		

The table details the direction of cytokine and chemokine concentration changes described in patients with specific mental disorders across all collected sources of the literature individually. ASD, autism spectrum disorder; BD, bipolar disorder; CCL, C-C motif ligand; CRP, C reactive protein; CXCL, C-X-C motif chemokine ligand; FEP, first episode psychosis; IFN-γ, interferon γ; IGF, insulin-like growth factor; IL, interleukin; IL-RA, interleukin receptor antagonist; MDD, major depressive disorder; NC, not changed; NGF, nerve growth factor; NT, neutrophin; OCD, obsessive-compulsive disorder; PTSD, post traumatic stress disorder; SCZ, schizophrenia; sIL-R, soluble interleukin receptor; sTNF-R, soluble tumor necrosis factor receptor; S100, S100 calcium-binding protein B; TGF-β, transforming growth factor β; TNF-α, tumor necrosis factor α; VEGF, vascular endothelial growth factor; ↑, increase; ↓, decrease**2**.

**Table 2:** Cytokine profiles in mental disorders.

	IL-1α	IL-1β	IL-2	IL-4	IL-5	IL-6	IL-8	IL-10	IL-12	IL-17	IL-18	TNF-α	IFN-γ	TGF-β	CRP	sTNF-R1	sTNF-R2	IL-1RA	sIL-6R	sIL-2R	CCL2	CCL11	CXCL10
MDD		NC	NC	NC	NC	↑	NC	NC	↑		↑	↑			↑			↑		↑			
BD		NC	NC	↑	NC	↑/NC	NC	↑				↑	NC			↑	NC	↑/NC	↑	↑		↑	↑
SCZ		↑	NC			↑	↑	↑	↑			↑	↑					↑	↑				
OCD		↓				NC						↑/NC											
PTSD		↑/NC		NC		↑		↑/NC				↑	↑		NC								
ASD	NC	↑		NC		↑	↑			NC		↑	↑								↑	↑	
SUIC			↓	↓		↑								↑									
FEP		↑	NC	NC		↑		NC		NC		↑	NC										

The table shows the leading direction of cytokine changes in individual mental disorders, i.e., their cytokine profile. The source literature on which it is based is detailed in **Table 1**. The direction of changes was defined based on the frequency of occurrence in the source literature. Each study was assigned 1 point, while meta-analyses were assigned 2 points. The leading direction of change was considered to be the one with the highest total points from the studies describing it. In cases of insufficient data, the leading direction of changes was not determined. ASD, autism spectrum disorder; BD, bipolar disorder; CCL, C-C motif ligand; CRP, C reactive protein; CXCL, C-X-C motif chemokine ligand; FEP, first episode psychosis; IFN-γ, interferon γ; IL, interleukin; IL-RA, interleukin receptor antagonist; MDD, major depressive disorder; NC, not changed; OCD, obsessive-compulsive disorder; PTSD, post traumatic stress disorder; SCZ, schizophrenia; sIL-R, soluble interleukin receptor; sTNF-R, soluble tumor necrosis factor receptor; TGF-β, transforming growth factor β; TNF-α, tumor necrosis factor α; ↑, increase; ↓, decrease.

## 3 Viruses involved in neuropsychiatric illness

### 3.1 Influenza virus

Influenza viruses, classified under the *Orthomyxoviridae* family, are single-stranded RNA viruses comprising three types: A, B, and C. It replicates in a cytolytic cycle, causing apoptosis in many types of cells. In humans, it usually causes symptoms such as fever, chills, joint and muscle pain, weakness, cough, nasal congestion, and light sensitivity. It can also lead to more serious complications, such as pneumonia or bronchitis, and sometimes even death. These more serious complications are mainly associated with influenza A and B. Of these, *influenza A virus* exhibits the highest genetic variability, with subtypes distinguished based on their glycoproteins, hemagglutinins (H) and neuraminidases (N). There are 18 hemagglutinin and 11 neuraminidase subtypes. The most prevalent subtypes of *influenza A virus* affecting humans are H1N1 and H3N2. Notably, *influenza A* was responsible for the 1918 “Spanish flu” pandemic and the 1957 “Asian flu” epidemic (Bouvier and Palese, 2008; Centers for Disease Control and Prevention, 2023). A limited number of influenza virus strains, primarily *influenza A virus*, are neurotropic, meaning they can infect and replicate in nervous system cells. A few autopsy studies have documented the presence of influenza virus and its antigens in the cerebrospinal fluid and brain tissue of patients who succumbed to severe influenza. The regions of the brain where the virus has been found include the mesencephalon, thalamus, cortex, and hippocampus (Franková et al., 1977; Ishigami et al., 2004; Hosseini et al., 2018). Non-neurotropic subtypes, like H1N1 and H3N2, can also cause CNS complications, likely through indirect activation of microglial cells by inflammatory factors entering the CNS from the periphery (Barbosa-Silva et al., 2018).

#### 3.1.1 Psychiatric complications of influenza

The psychiatric complications of influenza remain a topic of ongoing research and debate. Early 20th-century studies and more recent research from the early 21st century suggest an association between maternal influenza infection during the first trimester of pregnancy and a sevenfold increase in the risk of SCZ in offspring compared to the general population. However, these findings necessitate further validation in larger cohorts (Brown et al., 2004). The same researcher observed a twofold increased risk of SCZ in adult offspring of mothers who experienced upper respiratory tract infections during the second trimester, an association not found for infections in the first and third trimesters (Brown et al., 2000b). Conversely, no increased SCZ risk was reported in offspring of mothers infected during the 1957 influenza epidemic, although this study relied on self-reported data and did not measure antibody levels (Selten et al., 2010). Influenza infection during pregnancy has also been implicated in a nearly fourfold increased risk of BD in offspring (Parboosing et al., 2013). Moreover, maternal influenza infection has been linked to a fivefold higher risk of developing BD with psychotic features in offspring, without a corresponding increase in BD without psychotic features (Canetta et al., 2014). Patients with a history of mood disorders are significantly more likely to be seropositive for influenza A or B. Notably, seropositivity for *influenza B virus* is associated with a more than 2.5-fold increased risk of suicide attempts and psychotic symptoms during affective episodes, a relationship not observed for *influenza A virus* (Okusaga et al., 2011). Contrarily, a 1981 study involving 400 participants found no differences in influenza A or B antibody levels between patients with and without depression (Sinanan and Hillary, 1981). The impact of influenza infection on psychiatric disease risk remains inconclusive. Research focusing on maternal influenza infection during pregnancy suggests a potential link to SCZ, though the virus rarely crosses the placenta (Irving et al., 2000).

#### 3.1.2 Cytokine profile of influenza and other mechanisms of its influence on the CNS

The virus’s effects on the fetal CNS are likely indirect, possibly mediated by maternal IgG antibodies crossing the placenta and cross-reacting with fetal brain antigens via molecular mimicry (Brown et al., 2004). Another potential mechanism involves elevated maternal cytokine levels, their transplacental passage, and subsequent impacts on fetal brain development (Gilmore and Jarskog, 1997; Brown et al., 2004; Ratnayake et al., 2013). However, studies on cytokine transfer across the placenta are mixed. Some indicate limited transfer for cytokines such as IL-1, IL-4, IL-8, IL-10, IL-13, and TNF-α (Reisenberger et al., 1996; Zaretsky et al., 2004; Aaltonen et al., 2005; Lim and Kobzik, 2009), whereas IL-6 and IL-2 have been shown to cross the placenta (Zaretsky et al., 2004; Dahlgren et al., 2006; Ponzio et al., 2007). Additionally, the placenta itself may produce cytokines in response to maternal infection or elevated cytokine levels (Shimoya et al., 1999; Thiex et al., 2009). Placental immune cells continuously produce cytokines like IL-11, IL-17A, IL-17F, TGF-β, and VEGF, and in response to endotoxin or polyinosinic:polycytidylic acid (poly I:C), they produce IL-1, IL-6, IL-8, IL-10, and TNF-α (Urakubo et al., 2001; Gilmore et al., 2005; Ashdown et al., 2006; Pavlov et al., 2020). Influenza may also influence the development of affective disorders in adults through cytokines released during the immune response, such as interferons, TNF-α, IL-1β, and IL-6, which activate the kynurenine pathway, producing neurotoxic metabolites. Elevated levels of 3-hydroxykynurenine, quinolinic acid, kynurenine, and kynurenic acid are associated with depressive disorders and mania (Correia and Vale, 2022; Karimi et al., 2022; Lorkiewicz and Waszkiewicz, 2022). A rare complication of influenza in adults is influenza-associated encephalitis (IAE), which can be acute, subacute, or late-onset. Psychiatric complications of IAE, similar to other causes of encephalitis, may include AD, insomnia, mood disorders, and psychotic symptoms (Ishigami et al., 2004; Meijer et al., 2016; Butler et al., 2024).

The cytokine profile commonly described in influenza studies includes increased levels of C-C motif ligand 2 (CCL2), IL-8, C-X-C motif ligand 10 (CXCL10), IFN-α, -β, -γ, IL-1β, IL-6, TNF-α, and IL-18 (Chan et al., 2005; Liu et al., 2016; Betakova et al., 2017; Fiore-Gartland et al., 2017; Guo X zhi and Thomas, 2017; Gu et al., 2019; Turianová et al., 2019; Gu et al., 2021). Studies in mice have shown that higher, lethal doses of the virus, unlike lower doses, induce IL-4, IL-7, IL-10, IL-11, IL-12p40, IL-13, IL-15, and shift Th1 cell polarization towards Th2, with increased expression of M2 macrophages (Turianová et al., 2019). The most common cytokine changes in viral infections mentioned above, i.e., the cytokine profile, are visualized in **Table 3**. From this table, it can be observed that the cytokine profile during mild to moderate influenza infection closely resembles those found in SCZ, autism spectrum disorder (ASD), and first-episode psychosis (FEP). Severe infections shift the profile more towards SCZ and BD. While ASD is not typically associated with influenza in scientific research, SCZ and BD are, particularly in offspring of mothers infected during pregnancy. Although the passage of cytokines through the placenta is unlikely, they may induce changes in the hypothalamic-pituitary-adrenal (HPA) axis (Dunn et al., 1989; Dunn, 2000), metabolic pathways (tryptophan) (Holtze et al., 2008; Campbell et al., 2014) and immunological responses, potentially increasing the risk of these mental disorders.

**Table 3:** Cytokine profiles in mental disorders and viral infections.

	IL-1α	IL-1β	IL-2	IL-4	IL-5	IL-6	IL-8	IL-10	IL-12	IL-17	IL-18	TNF-α	IFN-γ	TGF-β	CRP	sTNF-R1	sTNF-R2	IL-1RA	sIL-6R	sIL-2R	CCL2	CCL11	CXCL10
MDD		**NC**	**NC**	**NC**	**NC**	**↑**	**NC**	**NC**	**↑**		**↑**	**↑**			**↑**			**↑**		**↑**			
BD		**NC**	**NC**	**↑**	**NC**	**↑/NC**	**NC**	**↑**				**↑**	**NC**			**↑**	**NC**	**↑/NC**	**↑**	**↑**		**↑**	**↑**
SCZ		**↑**	**NC**			**↑**	**↑**	**↑**	**↑**			**↑**	**↑**					**↑**	**↑**				
OCD		**↓**				**NC**						**↑/NC**											
PTSD		**↑/NC**		**NC**		**↑**		**↑/NC**				**↑**	**↑**		**NC**								
ASD	**NC**	**↑**		**NC**		**↑**	**↑**			**NC**		**↑**	**↑**								**↑**	**↑**	
SUIC			**↓**	**↓**		**↑**								**↑**									
FEP		**↑**	**NC**	**NC**		**↑**		**NC**		**NC**		**↑**	**NC**										
INFL		**↑**		**↑↑**		**↑**	**↑**	**↑↑**	**↑↑**		**↑**	**↑**	**↑**								**↑↑**		**↑**
HSV		**↑**				**↑**	**↑**	**↑/NC**	**↑/NC**			**↑**	**↑**										**↑**
CMV		**↑**				**↑**	**↑**	**↑**				**↑**	**↑/NC**										**↑**
EBV			**NC**	**NC**	**NC**	**↑**	**↑**	**↑**	**↓**		**↑**	**↑**	**↑**										
BoDV	**↑**			**↑**	**↑**	**↑**	**↑**	**↑**	**↑**		**↑**	**↑**	**↑**								**↑**		**↑**
RV																							
HIV		**↑**	**↓**	**↑**		**↑**	**↑**	**↑**				**↑**	**↓**								**↑**		
SARS-CoV-2		**↑**	**↑**	**↑↑**		**↑**	**↑**	**↑↑**	**↑**	**↑**	**↑↑**	**↑**	**↓**	**↑**	**↑**						**↑**		**↑**

This table compares the cytokine profiles of individual mental disorders with the cytokine profiles of viral infections described in this study to facilitate the comparison of similarities or differences between them. The cytokine profile of viral infections was constructed using the same method as that of the mental disorders. Due to the smaller amount of data on cytokine disturbances in individual viral diseases and the easier traceability of these data, the individual source literature references were not listed in a separate table but were mentioned in the main text. ASD, autism spectrum disorder; BD, bipolar disorder; BoDV, borna disease virus; CCL, C-C motif ligand; CMV, cytomegalovirus; CRP, C reactive protein; CXCL, C-X-C motif chemokine ligand; EBV, Ebstein-Barr virus; FEP, first episode psychosis; HIV, human immunodeficiency virus; HSV, herpes simplex virus; IFN-γ, interferon γ; INFL, influenza; IL, interleukin; IL-RA, interleukin receptor antagonist; MDD, major depressive disorder; NC, not changed; OCD, obsessive-compulsive disorder; PTSD, post traumatic stress disorder; RV, rubella virus; SARS-CoV-2, Severe Acute Respiratory Syndrome Coronavirus 2; SCZ, schizophrenia; sIL-R, soluble interleukin receptor; sTNF-R, soluble tumor necrosis factor receptor; TGF-β, transforming growth factor β; TNF-α, tumor necrosis factor α; ↑, increase; ↓, decrease; ↑↑, increase only in severe infections.

#### 3.1.3 Conclusions

Despite historical reports suggesting a link between influenza infection and the development of mental illnesses in adults, more recent research does not clearly support this association if the infection occurred during adulthood. However, prenatal exposure to influenza may influence the development of psychiatric conditions such as SCZ and BD. The cytokine profile observed during infection is broader than those associated with these specific diseases.

### 3.2 Herpes simplex virus

HSV, a member of the *Herpesviridae* family of double-stranded DNA viruses, is classified into two subtypes: HSV-1 and HSV-2. According to the World Health Organization (WHO), over 3.7 billion individuals under the age of 50 are infected with HSV-1, and nearly 500 million individuals aged 15-49 are infected with HSV-2 (Herpes simplex virus, 2023). While HSV-1 and HSV-2 are primarily responsible for labial and genital herpes, they are also implicated in encephalitis, meningitis, and neonatal HSV infection (Xu et al., 2019). Postmortem studies have detected HSV DNA in various brain regions, particularly in the frontal and temporal cortex (Olsson et al., 2016; Marcocci et al., 2020). The virus typically infects via oral-labial or sexual routes and replicates primarily in a cytolytic manner. Following the acute phase, HSV establishes latent infection in the dorsal root ganglia, with potential reactivation triggered by factors such as excessive sunlight exposure, psychological and physical stress, menstruation, fever, and surgical procedures (Steiner et al., 2007).

#### 3.2.1 Psychiatric complications of HSV

A 2009 study revealed a significantly higher prevalence of IgM anti-HSV antibodies in children with ASD compared to healthy children (65% vs. 17.5%). The levels of other antibodies, such as anti-EBV, -CMV, -measles, or -RV, remained unchanged across groups. Additionally, 96% of children with ASD and IgM anti-HSV exhibited IgG antibodies against brain tissue (including anti -amygdala, -caudate, -cerebellum, -medulla, -hippocampus, -commissure magnum, and -cortex) in their serum, compared to only 17% of children with ASD without IgM anti-HSV (Mora et al., 2009). The presence and titers of these antibodies correlated directly with ASD severity, suggesting a potential influence of HSV on ASD severity, independent of causation. Furthermore, elevated titers of anti-HSV-2 IgG antibodies in mid-pregnancy maternal plasma have been associated with an increased risk of ASD in male offspring. This association was not observed for HSV-1, CMV, or RV (Mahic et al., 2017). However, the methodology and reporting of these findings have been questioned (Magaret and Wald, 2017). Other studies have not corroborated higher seropositivity for HSV-1 or HSV-2 in children with ASD compared to healthy controls, irrespective of congenital or later-acquired infection (Gentile et al., 2014; Zappulo et al., 2018). HSV’s impact on depression has also been documented. HSV-2 exposure, confirmed by anti-HSV-2 IgG antibodies, was associated with a twofold increased risk of depression, a finding not observed for HSV-1 (Simanek et al., 2014; Gale et al., 2018). In contrast, patients with BD did not exhibit increased seropositivity for HSV-1 or HSV-2 (Tedla et al., 2011; Snijders et al., 2019). Interestingly, studies have demonstrated a strong association between HSV and the development of SCZ. Elevated levels of anti-HSV-2 IgG and IgM in pregnant mothers are significantly associated with an increased risk of psychotic disorders, including SCZ, in their offspring, whereas this association was not observed for HSV-1 (Buka et al., 2001; Buka et al., 2008; Mortensen et al., 2010; Arias et al., 2012). Herpesviruses may also be linked to cognitive disorders and the development of late-onset Alzheimer’s disease. HSV-1 infection has been associated with amnesia, semantic memory impairment, visual agnosia, and impaired executive function, primarily following HSV-induced encephalitis (Lin et al., 2002; Damiano et al., 2022).

#### 3.2.2 Cytokine profile of HSV and other mechanisms of its influence on the CNS

The data suggest that HSV’s potential impact is most pronounced concerning SCZ and, to a lesser extent, ASD and depression. The risk appears to be subtype-dependent, with HSV-2 being more significant than HSV-1, despite the latter’s higher detection rate in the brain. The exact mechanism by which HSV influences these disorders remains unclear, though its neurotropic properties and detection of its DNA in the CNS of asymptomatic patients are noteworthy (Lin et al., 2002; Marcocci et al., 2020). This implies that HSV may exert effects on the CNS even in the absence of overt infection signs. **Table 3** illustrates the cytokines most commonly associated with HSV infection, which include TNF-α, IL-6, IL-1β, IL-8, IFN-α, IFN-β, IFN-γ, CCL5, and CXCL10, with IL-10 and IL-12 appearing in later infection stages (Mikloska et al., 1998; Li et al., 2006; Melchjorsen et al., 2006; Stefanidou et al., 2013; Feng et al., 2021; Smith et al., 2022). This cytokine profile resembles those observed in SCZ and BD, although research on HSV-induced cytokines is limited and often fails to differentiate between HSV-1 and HSV-2, potentially leading to erroneous conclusions. If HSV is assumed to increase SCZ risk, one probable pathway involves maternal IL-8 elevation during pregnancy, previously linked to increased SCZ risk and CNS developmental anomalies associated with SCZ, combined with HSV’s direct CNS effects (Buka et al., 2001; Marcocci et al., 2020).

#### 3.2.3 Conclusions

Some studies suggest a potential link between HSV-2 infection during pregnancy and ASD occurrence in offspring, although this remains uncertain and warrants further investigation. HSV-2 may also be associated with depression and SCZ in adults, with prenatal virus exposure elevating the risk. The cytokine profile of HSV infection partially aligns with those in SCZ and BD, although discrepancies exist regarding the associated mental illnesses. Maternal IL-8 elevation during pregnancy could be a significant factor influencing SCZ and psychosis development in offspring.

### 3.3 Cytomegalovirus

The CMV belongs to the *Herpesviridae* family and primarily infects humans. In industrialized nations, 60-70% of adults may carry the virus, while in developing countries, infection rates can reach nearly 100%. Transmission occurs via contact with bodily fluids from an infected individual. Similar to HSV, replication occurs in a cytolytic cycle, and after the primary infection, the virus enters a latency phase. In immunocompetent adults, CMV rarely leads to severe complications. Most often, the infection is asymptomatic or can resemble mononucleosis, causing fever, fatigue, muscle aches, and enlarged lymph nodes. However, in immunocompromised individuals, particularly those with AIDS, it can cause severe complications, including encephalitis, as confirmed by the detection of its DNA in the autopsy examinations of the brains of deceased patients (Morgello et al., 1987; Achim et al., 1994; Nangle et al., 2018; Balegamire et al., 2022). CMV can also be transmitted congenitally. According to the Centers for Disease Control and Prevention (CDC), vertical transmission of CMV occurs in approximately 1 in 200 births in the United States and is the leading infectious cause of birth defects. Congenital CMV (cCMV) is asymptomatic in up to 95% of cases at birth but can later result in severe complications, such as deafness, delayed psychomotor development, vision loss, and epilepsy. These complications occur in 15-18% of infected children and result in mortality in 5-10% of affected newborns (Yow et al., 1988; Centers for Disease Control and Prevention, 2022). Thus, CMV-related complications are closely linked to the central nervous system (CNS) and may be associated with the development of mental disorders.

#### 3.3.1 Psychiatric complications of CMV

Studies investigating the impact of CMV on depression indicate that CMV infection alone does not increase its risk. However, among those infected, a higher anti-CMV titer is significantly correlated with an increased risk of developing depression (Phillips et al., 2008; Gale et al., 2018). Among seropositive individuals, an increase in anti-CMV antibody concentration by one unit raises the risk of depression by 26%, with those having the highest antibody levels exhibiting nearly a fourfold increased risk. The link between CMV and other affective disorders is less clear. While a large meta-analysis did not find an association between CMV seropositivity and BD, a case-control study of 495 individuals noted that BD patients had significantly higher anti-CMV IgG titers compared to healthy controls (Tedla et al., 2011). Moreover, BD patients often test seropositive for CMV and that group of patients have a higher incidence of mania with psychotic symptoms (Frye et al., 2019). Furthermore, BD patients with elevated mood display higher anti-CMV antibody titers comparing to euthymic patients, and the higher antibody titres are, the greater impulsivity is (Prossin et al., 2013; Prossin et al., 2015). Despite the pathogenetic similarities between BD and SCZ, the impact of CMV on SCZ is less definitive and requires more research. A 2012 meta-analysis found no association between CMV and SCZ, and a systematic review the same year reported no effect of maternal CMV infection on the risk of developing SCZ in offspring (Arias et al., 2012; Khandaker et al., 2013).

#### 3.3.2 Cytokine profile of CMV and other mechanisms of its influence on the CNS

CMV has the greatest impact on increasing the risk of affective disorders. This virus is neurotropic and appears to have an affinity for the limbic system, which dysfunction plays a major role in affective disorders. In cases of intrauterine infection, the virus seems to favor the ventricular regions of the brain. Changes are also found in the hippocampus and olfactory bulb (van den Pol et al., 1999; Krstanović et al., 2021). The immune response to CMV is also significant. Reactivation of CMV can lead to the production of pro-inflammatory cytokines and subsequent blocking of type I and II interferons, which are essential for inflammation induction and defense against viruses (Abate et al., 2004; Marshall and Geballe, 2009). CMV seropositivity is associated with an inversion of the CD4:CD8 cell ratio due to the accumulation of low-diversity CD8 clones (Pawelec et al., 2009). The transfer of maternal CD8+ T cells through the placenta contributes to perinatal brain damage due to intrauterine infection, so the increase in CD8+ cells associated with CMV infection may be crucial for the neurological and psychiatric complications of this infection (Lei et al., 2018). CMV also induces a strong immune response from glial cells, resulting in the production and release of TNF-α, IL-6, CCL3, CCL5, and CXCL10 from microglia, and IL-8 and MCP-1 from astrocytes (Lokensgard et al., 2002). Chronic CMV infection and viral reactivation can elicit a sustained immune response, evidenced by elevated titers of anti-CMV antibodies. This persistent immune activation, in conjunction with the virus’s affinity for limbic structures, particularly the hippocampus, and localized cytokine secretion, may contribute to the pathological damage observed in these neural regions. The most frequently described peripheral cytokines produced during CMV infection are IL-1β, IL-6, IL-8, IL-10, TNF-α, CXCL10, and CCL5 (Humar et al., 1999; Lokensgard et al., 2002; Compton et al., 2003; Zhang et al., 2008; Clement and Humphreys, 2019; Chinta et al., 2020; Bourgon et al., 2022). This cytokine profile aligns most closely with those of SCZ and BD (as shown in **Table 3**), although the primary mechanism of CMV’s action may be more related to the previously mentioned cytokines secreted during glial activation.

#### 3.3.3 Conclusions

The relationship between CMV and depression appears promising, with some evidence suggesting a link to BD. The cytokine profile of CMV infection aligns with BD, but not depression. Further research is needed to clarify CMV’s role in affective disorders and to identify any non-inflammatory mechanisms contributing to these diseases.

### 3.4 Ebstein-Barr virus

EBV, a representative of the *Herpesviridae* family, targets B lymphocytes and epithelial cells, replicating within them in a lytic cycle before establishing latent infections. The most common disease caused by EBV is infectious mononucleosis, which is characterized by symptoms such as fever, sore throat, swollen lymph nodes, fatigue, muscle and joint pain, and sometimes an enlarged spleen and a skin rash that occurs after the administration of ampicillin. However, the virus is also associated with more severe conditions, such as Hodgkin’s lymphoma and Burkitt’s lymphoma. It is estimated that approximately 90% of the human population is infected with this virus (Gequelin et al., 2011; Shi et al., 2021). During the transition from the latent to the lytic phase of replication, the virus can penetrate the CNS alongside infected lymphocytes, damage the blood-brain barrier (BBB), thereby increasing its permeability, and replicate within the CNS (Jha et al., 2015; Lupia et al., 2020). EBV also has the ability to directly infect nerve cells, which some authors link to neurodegenerative diseases such as Alzheimer’s disease, Parkinson’s disease, and multiple sclerosis (MS) (Jha et al., 2015; Lee et al., 2021; Zhang et al., 2022). In the context of MS, autopsy examinations of 101 patients revealed the presence of EBV in the brain in up to 90% of cases; however, these changes were not associated with any specific region of the brain (Hassani et al., 2018).

#### 3.4.1 Psychiatric complications of EBV

Despite the potential for EBV to influence the CNS, there is no conclusive evidence linking it to mental illnesses. A 2012 meta-analysis did not confirm an association between EBV and SCZ, but a 2019 study involving over 400 SCZ patients found that they had higher levels of antibodies to some, but not all, EBV proteins compared to a healthy control group (Arias et al., 2012; Dickerson et al., 2019). Studies on the association of EBV with depression are also inconclusive. A meta-analysis on the relationship between infectious agents and depression showed that EBV-positive patients are almost twice as likely to develop depression (Wang et al., 2014). Other studies failed to confirm these results, but the authors of the aforementioned meta-analysis point out that only a sufficiently large sample can reveal a relationship between EBV and depression (Amsterdam et al., 1986; Conejero-Goldberg et al., 2003). Interestingly, pregnant women with symptoms of depression are significantly more likely to show immunological markers of EBV reactivation compared to healthy pregnant women. In this context, the question arises as to whether the reactivation of EBV precipitates depression during pregnancy, or conversely, whether depression and the concomitant immunosuppression facilitate EBV reactivation (Haeri et al., 2011; Johnson et al., 2011). Evidence for the latter may be the fact that depressive symptoms in pregnant women at 33-34 weeks of gestation were associated with a three-fold increase in EBV reactivation, as assessed one week before delivery (Zhu et al., 2013). BD has also been investigated for an association with EBV, including cerebrospinal fluid testing for anti-EBV antibodies, but no association has been observed (Stich et al., 2015; Barichello et al., 2016; Snijders et al., 2019). Therefore, the relationship between BD and EBV seems unlikely at this point.

#### 3.4.2 Cytokine profile of EBV and other mechanisms of its influence on the CNS

The potential mechanism by which EBV might cause MDD-like disorders involves its ability to migrate to the CNS with infected B lymphocytes. The virus can replicate in the CNS and infect neuronal cells, leading to chronic inflammation and cytokine production within CNS structures, contributing to the development of mental disorders. Moreover, EBV attacks immune system, causing its disorders and dysregulation. It sensitizes the infected individual to other infections, activates the stress axis, and disrupts the cytokine balance, which collectively could lead to the development of MDD. Cytokine imbalances most commonly described in EBV infection include increased concentrations of TNF-α, IL-6, IL-8, IL-10, IFN-γ, CCL3, CCL4, and sometimes IL-18 and IL-1β. Some authors also note a lack of increase or a decrease in the concentration of IL-2, IL-5, IL-12, and TGF-β (Mosialos, 2001; Attarbaschi et al., 2003; Miyauchi et al., 2011; Wada et al., 2013; Coleman et al., 2015; Draborg et al., 2016; cui et al., 2017; Kianfar et al., 2022). The cytokine profile of EBV infection aligns most closely with the cytokine profile of SCZ, but studies of the relationship between these two diseases do not show any correlation (see **Table 3**).

#### 3.4.3 Conclusions

Although EBV is a widely prevalent virus with potentially serious health implications, its direct association with most mental disorders remains inconclusive, except for a possible link with depression that requires further investigation.

### 3.5 Borna disease virus

BoDV, a single-stranded RNA virus belonging to the *Bornaviridae* family, primarily infects horses, sheep, and cattle. This neurotropic virus induces Bornean disease in animals, colloquially referred to as sad horse disease. In animals, BoDV can cause fatal encephalitis and symptoms such as ataxia, anxiety, hyperactivity, aggression, and depressive-like behaviors (Carbone, 2001). The primary route of infection is through the nasal and olfactory epithelium, where the virus targets nerve cells and travels towards the central nervous system (CNS). BoDV replicates in a non-cytolytic cycle and does not exhibit classical cytopathic properties, allowing it to evade detection and establish latent infection in the CNS. In adults and adolescents, although rare, BoDV can cause severe encephalitis with high mortality. Several autopsy studies have detected BoDV RNA and antigens in the brains of infected patients (De La Torre et al., 1996; Klaus et al., 2018; Riccò et al., 2023).

#### 3.5.1 Psychiatric complications of BoDV

Until the mid-1980s, it was believed that BoDV only infected animals. However, in 1985, antibodies against BoDV were detected in 16 out of 979 examined psychiatric patients (Rott et al., 1985). Subsequent studies revealed the presence of anti-BoDV antibodies in 5% of psychiatric patients compared to 3% of surgical patients (Bechter, 2013). A 2014 meta-analysis, encompassing 15 studies on BoDV and depression, indicated that individuals with depression are 3.25 times more likely to be infected with BoDV (Wang et al., 2014). Additionally, a case-control study in Iranian patients with affective disorders found anti-BoDV antibodies in 29.5% of controls and 40.04% of patients, with the highest seropositivity observed in patients with BD at 45.03% (Mazaheri-Tehrani et al., 2014). Recent research also demonstrated that antiretroviral therapy with amantadine significantly alleviated depressive symptoms and reduced suicidal behavior in BoDV-seropositive patients (Dietrich et al., 2020). According to a 2012 meta-analysis, there is a notable association between BoDV infection and the incidence of SCZ (Arias et al., 2012). However, the retrospective nature of most studies complicates the assessment of whether BoDV infection genuinely increases the risk of developing mental disorders, as the infection may precede or coincide with the onset of these disorders.

#### 3.5.2 Cytokine profile of BoDV and other mechanisms of its influence on the CNS

The neurotropic nature of BoDV and the symptomatic manifestations in animals suggest a potential role for BoDV in the pathogenesis of human mental disorders. BoDV exhibits a high affinity for the limbic system, potentially damaging its structures, which is characteristic of affective disorders (Gonzalez–Dunia et al., 1997; Rubin et al., 1999; Ovanesov et al., 2008). Infection during the developmental stages of the CNS may lead to neuroanatomical damage and impairments in neuroplasticity and neural conduction (Carbone, 2001). Neuronal infection by BoDV induces significant increases in DNA double-strand breaks (DSBs), adversely affecting neurotransmission regulation and cognitive functions. Infected cells exhibit reduced expression of surface NMDAR receptors and damage to the GluN2A subunit, resulting in disrupted glutamatergic transmission and decreased spontaneous electrical activity in neuronal networks (Marty et al., 2021). Glutamatergic pathway disturbances are critical in the pathophysiology of SCZ, MDD, and BD. Furthermore, cytokines such as IL-5, IL-6, IL-9, IL-10, IL-12p40, IL-13, IL-18, TNF-α, and IL-1α are expressed in BoDV-infected brains, with their concentrations correlating with neurological symptom severity. BoDV also induces IFN-γ production in astrocytes and microglia and the secretion of chemokines such as CCL-2, CCL-5, CXCL-10, and IL-8 (Rauch et al., [[NoYear]]; Gonzalez–Dunia et al., 1997). During the transition from acute to chronic infection, a shift from a Th1 to a Th2 immune response occurs, accompanied by increased IL-4 secretion (Hatalski et al., 1998). The cytokine profile associated with BoDV infection is broad and non-specific, with several changes overlapping with cytokine alterations observed in SCZ and MDD, though some do not align with the cytokine profiles of these disorders, which may be seen in **Table 3**.

#### 3.5.3 Conclusions

There is evidence suggesting that BoDV may play a role in the pathogenesis of certain mental disorders. Given the current state of knowledge, its association with affective disorders appears most likely, which aligns with the symptoms it causes in animals. However, the cytokine profile of the infection is quite non-specific, although its mechanism of action in the brain would support the occurrence of such diseases. Further research is necessary to definitively confirm these associations.

### 3.6 Rubella virus

The RV is a single-stranded RNA virus belonging to the *Matonaviridae* family, with humans as its sole host. Transmission occurs via respiratory droplets and transplacental routes. Regarding its replication, RV is typically described as a non-cytolytic virus, but it is cytopathic, inducing apoptosis in cells or inhibiting cell division. Postnatal infections are predominantly asymptomatic or result in a mild clinical course, presenting with symptoms such as rash, fever, swollen lymph nodes, headache, sore throat, joint and muscle pain, and less commonly, conjunctivitis. However, prenatal infections can lead to congenital rubella syndrome (CRS), characterized by significant anomalies including the classical CRS triad of cataracts, cardiac defects, and sensorineural deafness. There are limited post-mortem studies on RV; however, several autopsy reports of fatal CRS cases have identified RV antigens in brain tissues (Lazar et al., 2016). Infection may also result in stillbirth or spontaneous miscarriage, with the highest risk of complications occurring during the first trimester (Parkman, 1996; Popova et al., 2022).

#### 3.6.1 Psychiatric complications of RV

Historical data from 1971 revealed that 125 out of 243 children with CRS exhibited mental disorders, predominantly intellectual disability. Notably, 10 children were diagnosed with ASD, and 8 exhibited partial autistic symptoms, indicating a 200 times higher incidence compared to the general population. This led to the hypothesis that ASD may have organic etiologies, including RV infection (Chess, 1971; Chess, 1977). A 2017 study assessing anti-RV IgG levels in the mid-pregnancy blood samples of 442 mothers of children with ASD found no significant association between antibody levels and the occurrence of ASD in their offspring (Mahic et al., 2017). Mathematical modeling has estimated that RV vaccination prevented approximately 16,000 CRS cases between 2001 and 2010, which corresponds to the prevention of around 1,228 ASD cases (Berger et al., 2011). In developed countries, RV vaccination is widespread; however, in certain regions, vaccination is not yet standardized, with RV affecting about 5% of pregnant women globally. The RV may also contribute to other mental disorders beyond ASD. Intrauterine RV exposure, particularly during the first trimester, is associated with a five-fold increased risk of non-affective psychosis in adulthood. Additionally, individuals prenatally exposed to RV have a higher likelihood of developing SCZ spectrum disorders later in life (Brown et al., 2000a; Brown et al., 2001). Reports of psychiatric symptoms linked to adult RV infections are scarce. Nevertheless, CRS can lead to the development of panencephalitis in later life, even adulthood, potentially exacerbating neurological deficits and resulting in epileptic seizures, intellectual decline, ataxia, and spasticity (Townsend et al., 1975).

#### 3.6.2 Cytokine profile of RV and other mechanisms of its influence on the CNS

The mechanism through which RV may contribute to the development of these disorders involves its ability to cross the placenta and directly affect fetal brain tissue. The virus primarily targets microglial cells, which become susceptible to infection due to factors released by other brain cells. Microglial cells cannot be infected in the absence of those factors. Infected microglia secrete interferon, inducing a response from other cells and leading to chronic inflammation that disrupts CNS development (Popova et al., 2022). In the developing brain, this inflammation causes circulatory disturbances and subsequent ischemia, resulting in damage. The RV also inhibits mitosis, leading to reduced brain growth, hypocellularity, and delayed myelination due to impaired oligodendrocyte replication (Brown et al., 2001). While comprehensive studies on RV-related cytokine disturbances are limited, which makes the analysis of the cytokine profile impossible (which is the reason why the cytokine profile of RV infection is missing in **Table 3**), it is known that the primary cellular response involves interferon production. RV infection induces the secretion of type I and III interferons (e.g., IFN-α, IFN-β, IFN-λ) (Schilling et al., 2021). Overproduction of IFN-α/β in the brain leads to dendritic loss, reduced neurogenesis, decreased neurotrophic signaling, and impaired glutamatergic signaling in hippocampal and pyramidal neurons. These effects contribute to deficits in long-term potentiation and inhibition of angiogenesis (Viengkhou and Hofer, 2023). Chronic elevation of IFN-I levels in the CNS, referred to as cerebral interferonopathy, may also occur. Neuropsychiatric complications associated with interferon are well-documented, with patients often developing depression, fatigue, personality changes, cognitive disorders, psychosis, or mania, which typically resolve upon discontinuation of interferon therapy (Onyike et al., 2004; Cheng et al., 2009; Hoyo-Becerra et al., 2014; Pinto and Andrade, 2016; Su et al., 2019). Most of the available studies on interferon in mental disorders described the level of type II interferon, i.e., IFN-γ, as detailed in **Tables 1**, **2**. All interferons have similar effects; however, IFN-γ most strongly activates macrophages.

#### 3.6.3 Conclusions

There is evidence to suggest that CRS may be associated with mental disorders, although such evidence is scarce. Mentions of psychiatric complications from RV infection in adults are also rare, making such a connection doubtful. However, the RV’s life cycle and mechanisms of action in the brain seems conducive to the occurrence of mental disorders. Therefore, further research is needed to confirm or refute the association of this infection with mental illnesses.

### 3.7 Human immunodeficiency virus

HIV is a single-stranded RNA virus belonging to the *Retroviridae* family. According to World Health Organization (WHO) statistics, at the end of 2022, approximately 39 million people globally were living with HIV, and approximately 40 million have died as a result of the infection since its identification (HIV and AIDS, 2023). The virus is transmitted through human body fluids and attacks the host’s immune system, particularly cells expressing the CD4 receptor, including T helper cells, macrophages, dendritic cells, and astrocytes. It is not classically cytolytic, but it has cytopathic properties and slowly destroys immune system cells (Seitz, 2016). Until recently, HIV infection was nearly synonymous with premature death; however, the advent of effective antiretroviral therapies has significantly altered this prognosis (Eggleton and Nagalli, 2023). Untreated HIV infection leads to a gradual decrease in CD4+ cell counts, culminating in the development of acquired immunodeficiency syndrome (AIDS). The progression to AIDS is marked by numerous non-specific symptoms, diseases, and opportunistic infections, such as aseptic meningitis, encephalitis, peripheral neuropathy, herpes zoster, and HIV-associated encephalopathy (Seitz, 2016; Justiz Vaillant and Naik, 2023). HIV exhibits neuroinvasiveness by replicating in the brain’s microglia and macrophages. It is also described as neurotropic, although this property is contested by some researchers. Nevertheless, it has been shown to have a predilection for specific brain regions such as the fronto-cortical regions, basal ganglia, caudate, putamen, globus pallidus, and substantia nigra, as observed in autopsy studies (Ghorpade et al., 1998; Kumar et al., 2011). Neurological and neuropsychiatric disorders can manifest at any stage of infection, with approximately 50% of adults with AIDS experiencing neurological complications. These complications are collectively referred to as HIV-associated neurocognitive disorders (HAND), encompassing asymptomatic neurocognitive impairment (ANI), mild neurocognitive disorder (MND), and HIV-associated dementia (HAD). Despite the effectiveness of highly active antiretroviral therapy (HAART) in reducing the incidence of HAD, the prevalence of ANI and MND continues to increase, potentially due to prolonged survival, accumulated neurological damage, and chronic inflammation. It is noteworthy that the positive effects of HAART on the peripheral immune system may not fully extend to the CNS (Rao et al., 2014).

#### 3.7.1 Psychiatric complications of HIV

A meta-analysis conducted in 2014 indicated that 39.1% of individuals living with HIV experience depression (Uthman et al., 2014). HIV-positive individuals are 2 to 7 times more likely to meet the criteria for MDD compared to uninfected populations (Satz et al., 1997; Ciesla and Roberts, 2001). The stigma and fear associated with HIV infection, alongside the activation of the stress axis, may further exacerbate the risk of depression. Moreover, the viral mechanism, particularly the exposure of the brain to the viral Tat protein, has been shown to initiate a cytokine signaling pathway leading to depression-like sickness behavior in mouse models (Lawson et al., 2011). Additionally, certain antiretroviral medications, such as efavirenz, have been linked to an increased incidence of depressive symptoms, which may be due to efavirenz enhancing the activity of tryptophan-2-3-dioxygenase and redirecting tryptophan to the kynurenine pathway, thereby increasing the levels of its neurotoxic metabolites and decreasing serotonin levels in the brain (Munjal et al., 2017; Checa et al., 2020).

In addition to MDD, HIV-positive patients exhibit a higher prevalence of psychosis. HIV-related psychosis can be categorized as primary or secondary. Primary psychosis occurs in the absence of any HIV-related neurological disorder or acute metabolic dysfunction, directly attributed to the viral infection itself. Conversely, secondary psychosis is associated with opportunistic brain infections or metabolic encephalopathy related to respiratory, hepatic, or renal failure (Alciati et al., 2001). A literature review from 1991 documented cases of 31 HIV-infected patients who developed psychotic symptoms such as delusions, hallucinations, bizarre behavior, mood disorders, and cognitive disorders without any other identifiable cause (Harris et al., 1991). A casuistic case report described psychosis as the initial symptom of HIV infection, which was resistant to neuroleptic treatment but subsided following the introduction of HAART (Hashimoto et al., 2006). The incidence of new-onset psychosis in HIV patients is estimated at 3.7%, with those not receiving antiretroviral therapy being approximately 20 times more likely to develop psychotic disorders compared to those on such therapy (de Ronchi et al., 2000). Interestingly, a study comparing HIV-positive patients with and without psychosis found no significant differences in CD4+ count, brain MRI, or CSF, with the only distinguishing factor being a higher prevalence of psychoactive substance use in the psychotic group (Sewell et al., 1994). A 2013 review noted that 3% to 23% of psychotic patients were HIV-positive, and the incidence of newly developed psychosis in HIV-positive patients ranged from 0.23% to 15.2% (Nebhinani and Mattoo, 2013). Given the high prevalence of psychotic disorders among HIV-positive individuals, clinicians should remain vigilant for psychotic symptoms and conduct thorough assessments of risk factors, including substance abuse and previous psychiatric treatment. HIV infection should be considered in all patients presenting with new-onset psychosis, particularly those with late-onset or atypical presentations and multiple HIV risk factors.

#### 3.7.2 Cytokine profile of HIV and other mechanisms of its influence on the CNS

A full description of the impact of HIV on the CNS and the pathophysiology of the infection extends beyond the scope of this study. However, it is essential to note that HIV’s neuroinvasiveness is based on the migration of virus-infected monocyte-derived macrophages (MDM) into the brain. These macrophages produce inflammatory factors and chemokines that attract additional infected immune cells to the brain. The infection spreads to cells such as perivascular macrophages and microglia, and the inflammatory factors they produce further damage the BBB and increase its permeability. While neurons are not directly infected by HIV, they express receptors such as CCR5, CXCR4, and NMDAR, which are sensitive to viral proteins like gp120 or Tat, as well as to inflammatory factors such as TNF-α, IL-1β, and protein oxidation products. Elevated levels of TNF-α can inhibit glutamate uptake by astrocytes, resulting in glutamate accumulation in the extracellular space and subsequent excitotoxicity (Ghorpade et al., 1998; Dunfee et al., 2006; Rao et al., 2014).

SPECT studies have revealed ischemic changes in the brains of HIV-positive patients with psychosis that are not visible on conventional CT and MRI scans and are absent in psychotic HIV-negative patients. This suggests that psychosis in HIV-infected individuals may be associated with HIV encephalopathy (Masdeu et al., 1991). However, autopsy studies of psychotic HIV-positive patients have indicated that HIV may not be detectable in the CNS of some patients, suggesting that the initial viral stimulus can trigger a cytokine cascade that continues even in the absence of active viral replication (Sewell et al., 1994). Mental disorders in HIV patients may also result from opportunistic infections such as cerebral toxoplasmosis or be iatrogenic, due to treatments with medications like zidovudine or abacavir (Foster et al., 2003; Navinés et al., 2012; Abers et al., 2014; Torrey, 2022). Additionally, the CNS may serve as an independent site of viral replication, with CNS viral loads not always correlating with peripheral viremia. The varying degrees of antiretroviral drug penetration into the brain imply that effective peripheral viral suppression does not necessarily ensure CNS clearance (Munjal et al., 2017).

Cytokines produced in response to HIV infection include TNF-α, IL-1β, IL-4, IL-6, IL-8, IL-10, and IFN-α, while the production of IL-2 and IFN-γ is inhibited. Certain cytokines, such as IL-1, IL-6, and TNF-α, stimulate HIV replication *in vitro*, whereas others like IFN-α, IFN-β, IL-10, IL-13, and IL-16 inhibit it. IFN-γ and IL-4 exhibit mixed effects on HIV replication (Emilie et al., 1994; Kedzierska and Crowe, 2001; Le Saout et al., 2012; Osuji et al., 2018; Szymańska et al., 2022). The number of CD4+ and CD8+ cells does not correlate with interleukin concentrations, indicating that high levels of inflammatory factors may influence the development of mental disorders at any stage of the disease (Mlambo et al., 2019). **Table 3** shows that the cytokine profile of HIV infection most closely resembles the cytokine profile of SCZ, but not that of MDD, which is the leading mental disorder among infected individuals. While HIV infection is not associated with an increased risk of developing SCZ, the cytokine profile of the infection may partially explain the occurrence of psychotic symptoms among infected patients.

#### 3.7.3 Conclusions

Summarizing, there is evidence supporting the increased prevalence of mental and cognitive disorders among HIV patients. However, the cytokine profile of this infection does not completely align with the cytokine profile of these disorders. Therefore, the relationship between HIV and mental disorders is most likely multifactorial, and its understanding requires further detailed investigation.

### 3.8 Severe acute respiratory syndrome Coronavirus 2

SARS-CoV-2 is a recently identified virus whose emergence and rapid spread led to the 2020 pandemic. As of now, over 770 million infections have been confirmed, and almost 7 million people have died due to the virus, with new infections continuing to occur (WHO coronavirus (COVID-19) dashboard). This virus belongs to the *Coronaviridae* family, which also includes SARS-CoV and MERS-CoV. SARS-CoV-2 is a single-stranded RNA virus, transmitted via respiratory droplets. It is considered to be cytolytic and cytopathic. It causes pneumonia and acute respiratory distress syndrome, as well as milder forms of the disease with flu-like symptoms, loss of smell and taste, or sometimes gastrointestinal symptoms (Hu et al., 2021).. In 2020-2021, the main variants identified in infected individuals were alpha, beta, gamma, delta, and omicron (Carabelli et al., 2023). SARS-CoV-2 uses the angiotensin-converting enzyme 2 (ACE2) receptor to gain entry into cells, while the transmembrane serine protease 2 (TMPRSS2) facilitates the production of the viral S protein, which binds to ACE2 (Hoffmann et al., 2020). It mainly infects multiciliated cells in the nasopharynx or trachea, supporting cells in the olfactory mucosa of the nose, and alveolar cells in the lungs (Lamers and Haagmans, 2022). Studies in transgenic mice have demonstrated that the virus can enter the CNS via olfactory nerves, with high ACE2 expression detected in the mouse olfactory bulb, but ACE2 expression has not been confirmed in the human olfactory bulb and olfactory neurons. Instead, in humans, high ACE2 expression is found in the piriform cortex, which connects directly to the olfactory bulb, as well as in the choroid plexus, paraventricular nuclei of the thalamus, middle temporal gyrus, and posterior cingulate cortex (Chen et al., 2020; Chen et al., 2021). The potential for viral spread from supporting cells to neurons remains unclear, and requires further exploration. An autopsy study indicated the presence of the virus in the olfactory mucosa and neuroanatomical regions receiving olfactory tract projections, suggesting potential neuroinvasion of SARS-CoV-2 via axonal transport (Meinhardt et al., 2021). Additionally, some evidence points that SARS-CoV-2 may use neuropilin 1 (NRP1) to infect and that it can be modified by proteins such as cathepsin L (CTSL) and furin, which are expressed at much higher levels than ACE2 in the CNS and olfactory bulb (Burks et al., 2021; Mayi et al., 2021). There are also hypotheses suggesting that the virus may enter the brain through the bloodstream across the BBB barrier, the permeability of which increases due to a cytokine storm or interaction of the virus with the ACE2 receptor on endothelial cells capillaries included in the barrier (Davies et al., 2020).

#### 3.8.1 Psychiatric complications of SARS-CoV-2

One of the largest studies on psychiatric complications of COVID-19 was conducted using electronic medical records databases. The findings indicate that contracting COVID-19, compared to influenza and other infectious respiratory diseases, doubles the risk of developing a first episode of any mental illness. The severity of COVID-19 correlates with a likelihood of psychiatric complications, especially in most severe form of infection such as encephalopathy, where the risk of FEP is fivefold higher compared to COVID-19 patients without encephalopathy. Compared to influenza, SARS-CoV-2 infection is more likely to induce mood disorders (HR=1.79), AD (HR=1.78), psychotic disorders (HR=2.16), and insomnia (HR=1.92). The increased risk of disorders such as affective, anxiety, and insomnia persists for up to 90 days after infection. The cumulative incidence of AD aligns with that of the general population after those 90 days, but for depressive disorders, it takes 457 days. Conversely, the risk of cognitive and psychotic disorders remains elevated for more than two years post-infection, with their cumulative incidence also remaining higher (Taquet et al., 2021b; Taquet et al., 2021a; Taquet et al., 2022). These results are of high clinical importance as they mean that COVID-19 patients should remain under psychiatric monitoring for over two years post-illness, and their deteriorated cognitive functions for a long time may affect the quality of their life and cause difficulties in functioning in society and at work. The research group led by Taquet et al (Taquet et al., 2022). also compared which of the common SARS-CoV-2 variants caused the greatest increase in the risk of mental illness. Their findings, summarized in **Table 4**, indicate that the delta variant, identified in September 2020 and responsible for most cases in 2021, is associated with the highest increase in risk for the aforementioned disorders.

**Table 4:** Comparison of the hazard ratio of specific mental disorders depending on the variant of the SARS-CoV-2 virus.

Type of disorder	Variant α	Variant δ	Variant o
Anxiety disorder	HR 0.99	HR 1.10	HR 1.04
Mood disorder	HR 1.04	HR 0.99	HR 1.20
Cognitive impairment	HR 0.93	HR 1.13	HR 0.94
Psychotic disorder	HR 0.94	HR 1.15	HR 0.96
Insomnia	HR 0.94	HR 1.19	HR 0.95

Based on Taquet et al. ‘Neurological and psychiatric risk trajectories after SARS-CoV-2 infection: an analysis of 2-year retrospective cohort studies including 1 284 437 patients’.

Interestingly, a study using control groups from pre-COVID-19 times compared to a control group during the COVID-19 pandemic found no significant difference in the risk of mental disorders between the two groups. This suggests that the pandemic itself, including associated stress and social isolation, may not significantly influence the development of mental disorders, highlighting the importance of actual SARS-CoV-2 infection (Xie et al., 2022). However, a 2020 meta-analysis on the mental health of the general population indicated that up to one-third of individuals not directly affected by COVID-19, but living through the pandemic, experienced AD and depression. This observation suggests an impact of pandemic-related factors on mental health but does not fully exclude the direct effects of the virus, especially since the meta-analysis did not include pre-COVID control groups or account for mild or asymptomatic infections (Salari et al., 2020). In addition to depressive and AD, COVID-19 may also precipitate mania, as we observed in our previous study, where we collected reports of mania in 36 patients where COVID-19 was the most likely cause (Lorkiewicz and Waszkiewicz, 2022). Contrary to previous findings, a large 2023 study showed that SARS-CoV-2-infected patients do not have an increased risk of mental disorders within one year of hospitalization compared to patients hospitalized due to sepsis or influenza. The risk was increased only in the first 30 days post-hospitalization. However, the likelihood of other complications, such as venous thromboembolism, remained elevated for over a year (Quinn et al., 2023). Although the increased risk of neuropsychiatric disorders among COVID-19 patients appears to decrease relatively quickly, the long-term mental health effects of the infection remain uncertain. One of the distant and difficult to observe may be the effect of prenatal exposure to SARS-CoV-2.

#### 3.8.2 Cytokine profile of HIV and other mechanisms of its influence on the CNS

COVID-19 often involves a cytokine storm, characterized by uncontrolled, self-propelling overproduction and secretion of pro- and anti-inflammatory cytokines. The most common cytokine disturbances in this group of patients include elevated levels of IL-1β, IL-2, IL-4, IL-6, IL-7, IL-8, IL-10, IL-12, IL-17, IL-18, TNF-α, CRP, CCL2, CXCL-10, CCL3, TGF-β, as well as variable levels of IFN-γ. The production of type I interferons and the antiviral response are impaired. The virus causes a reduction in CD4+ and CD8+ T cells, which correlates with COVID-19 severity. In the initial stage of the disease, the immune response is mediated by Th1 cells, leading to constant activation and polarization of monocytes and pro-inflammatory cytokine production. In later stages, with reduced CD4+ and CD8+ T cells, a Th2 response predominates, producing cytokines such as IL-4, IL-5, and IL-13, which is associated with worse prognosis. Severe COVID-19 may also involve Th17 activation and production of IL-17, IL-21, and IL-22 (Hasanvand, 2022; Lamers and Haagmans, 2022; Lorkiewicz and Waszkiewicz, 2022; Montazersaheb et al., 2022). **Table 3** shows the extensive range of cytokine disturbances in COVID-19. Additionally, coagulation disorders and vascular endothelium damage occur, contributing to CNS vascular lesions and increased BBB permeability, exacerbating CNS damage and the risk of mental disorders (Lamers and Haagmans, 2022). In addition to neuroinvasiveness, SARS-CoV-2 exhibits neurotropism, although research in this area is inconclusive. Human brain autopsy studies have demonstrated the presence of viral antigens in parenchyma and cortical neurons, as well as viral RNA in the substantia nigra. Animal studies have shown susceptibility to the virus in cells such as dopaminergic neurons, microglia, and astrocytes. However, most studies indicate that SARS-CoV-2 replication in these cells is inefficient. Nevertheless, infected cells may undergo changes in their functioning and cellular processes (Bauer et al., 2022). The ability of SARS-CoV-2 to affect the CNS is also evident through its neurovirulence, i.e., its capacity to cause CNS pathology regardless of its ability to penetrate or infect CNS cells. This influence is mediated by cytokine and immunological dysregulation, oxidative stress, hypoxia, and prothrombotic potential (Bauer et al., 2022; Lamers and Haagmans, 2022)

Cytokine disturbances in COVID-19 most closely resemble those observed in SCZ, BD, and FEP. However, the breadth of these disturbances makes it difficult to into a single disease. Additionally, the severe course of COVID-19 is connected to a whole range of additional changes that may influence the development of mental diseases. These changes include a marked increase in the production of free radicals, leading to protein and lipid oxidation, neurotransmission disturbances, hypothalamic-pituitary-adrenal (HPA) axis dysregulation, alterations in hormonal balance, electrolyte imbalances, and hypoxia.

#### 3.8.3 Conclusions

In summary, SARS-CoV-2 infection is associated with an increased risk of developing mental illnesses, although this elevated risk appears to be limited to a specific period of time for most of these disorders. The cytokine profile of the infection is broad and nonspecific, making it impossible to link it to any particular mental disorder. The long-term psychiatric complications of the infection remain unknown, and the complexity of the pathophysiological changes occurring during the infection, along with other risk factors, necessitates further in-depth research to fully understand the underlying mechanisms and long-term impacts of COVID-19 on mental health.

## 4 Discussion

Prenatal exposure to certain viruses may increase the risk of SCZ, BD, or ASD. However, this conclusion is tentative due to the reliance on archival data and interviews instead of laboratory indicators (Brown and Derkits, 2010). Most viruses do not cross the placenta directly, implying an indirect influence on mental illness development through changes in maternal cytokine concentrations. Although only IL-6 and IL-2 are proven to cross the placenta, the placenta can produce cytokines in response to pathogens or increased maternal cytokine levels (Reisenberger et al., 1996; Shimoya et al., 1999; Zaretsky et al., 2004; Aaltonen et al., 2005; Dahlgren et al., 2006; Ponzio et al., 2007; Lim and Kobzik, 2009; Thiex et al., 2009; Pavlov et al., 2020). Maternal antibodies, like anti-influenza IgG, can also penetrate the placenta and affect fetal brain development (Brown et al., 2004). Pregnancy necessitates extensive physical, biochemical, and metabolic changes, many of which are mediated by cytokines. Initially, the pregnancy is characterized by a dominance of pro-inflammatory cytokines. Subsequently, there is a shift towards anti-inflammatory cytokines, facilitating the development of maternal-fetal symbiosis. In the final phase, the interaction of prostaglandins and primarily inflammatory cytokines orchestrates the final stages of fetal development and the initiation of labor (Dutta and Sengupta, 2017). Any cytokine imbalance during infection can lead to placental dysfunction, immunological changes, fetal defects, and neurodevelopmental issues (Meyer et al., 2008; Thaxton and Sharma, 2010; Yockey and Iwasaki, 2018). Beyond prenatal exposure, viruses can exhibit neuroinvasiveness, neurotropism, or neurovirulence, impacting the CNS in adults. While first two characteristics allow direct CNS infection, neurovirulence, an indirect mechanism, might increase mental disorder risk through cytokine disturbances (Bauer et al., 2022). Most viruses discussed here exhibit some degree of neurotropism, though the capacity of SARS-CoV-2 and HIV to infect neural cells remains a subject of debate (Ghorpade et al., 1998; Malik and Eugenin, 2016; Meinhardt et al., 2021). Non-neurotropic viruses include certain influenza strains, such as H1N1 and H3N2. If the influence of viruses on the development of mental disorders were solely dependent on their direct effects on the CNS and their neurotropism, patients infected with viruses like HIV, SARS-CoV-2, H1N1 and H3N2 would exhibit mental disorders much less frequently than those infected with highly neurotropic viruses like EBV. However, empirical data indicate the opposite. Thus, neurotropism alone does not account for the psychological complications of viral infections. Neurovirulence, along with cytokine disturbances, appears to play an equally or more significant role in this process.

Cytokine disturbances, especially increases in IL-6 and TNF-α, are common in both mental illnesses and viral infections, but are very unspecific (see **Table 3**). These can activate indoleamine 2,3-dioxygenase (IDO), disrupt hippocampal neurogenesis, induce apoptosis, increase blood-brain barrier (BBB) permeability, and stimulate amygdala activity (Lorkiewicz and Waszkiewicz, 2021). Elevated IL-8 and IL-10 are linked to SCZ and BD, though not all viral infections with these cytokine increases lead to corresponding mental disorders. Therefore, their expected impact is not the actual one. Nonetheless, the combination of IL-8 and IL-10 is often seen in diseases with a higher risk of psychotic symptoms (e.g., SCZ or BD). Viral infections frequently elevate IL-1β, which is also increased in SCZ and FEP. Viruses like influenza, CMV, BoDV, HSV-2, SARS-CoV-2, HIV, and EBV can influence the development and severity of depression, but their cytokine profiles do not entirely match those seen in MDD. For visual representation of these changes and similarities, and differences in cytokine profiles, refer to **Tables 1**, **3**. Even within a single virus species, the risk of mental disorders can vary by subtype, such as between HSV-1 and HSV-2. Additionally, co-occurrence of mental disorders (e.g., SCZ with depressive episodes) can transform immune responses (e.g., Th2 to Th1), complicating the assessment of cytokine profiles (Waszkiewicz, 2023).

**Figure 1:**
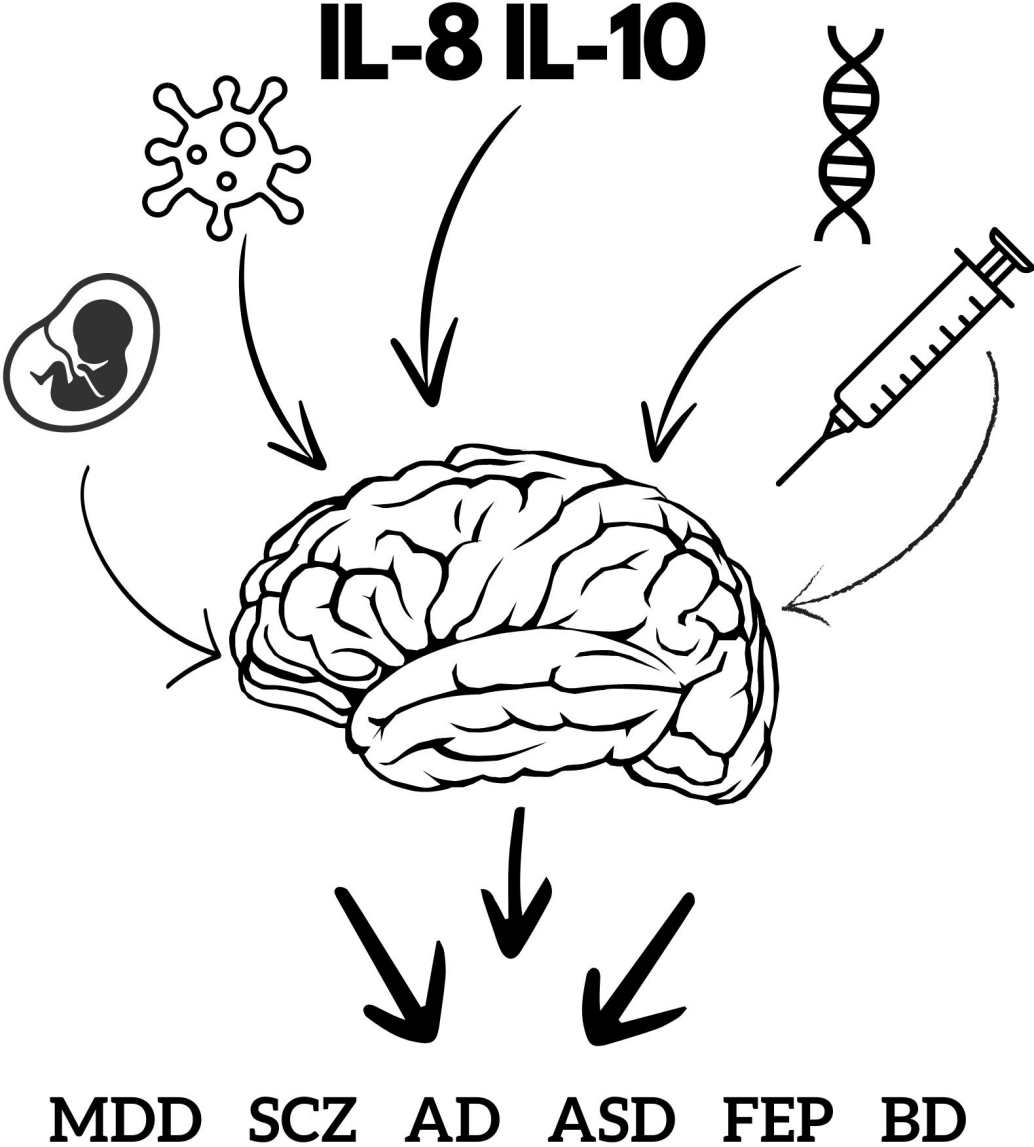
Factors affecting mental health. This figure schematically represents factors involved in the development of mental disorders, illustrating that cytokines are only part of a larger whole. The fetus represents the age and developmental stage at which viral infection occurred. The virus diagram represents the type of virus and neurotoxic viral proteins. The label IL-8 and IL-10 represents the involvement of cytokines and other inflammatory factors. DNA represents genetic factors and family history. The syringe represents a history of drug abuse. AD, anxiety disorder; ASD, autism spectrum disorder; BD, bipolar disorder; FEP, first episode psychosis; MDD, major depressive disorder; SCZ, schizophrenia.

Other viral mechanisms may also contribute to the development of mental diseases. Latent infections can cause recurrent CNS disorders and BBB damage when the virus exits latency (Steiner et al., 2007; Jha et al., 2015; Suzich and Cliffe, 2018; Lupia et al., 2020). Viral infections can induce autoantibody production, such as HSV-related antibodies against brain tissue (Mora et al., 2009). Molecular changes, such as BoDV-induced DNA strand breaks or RV-induced mitosis disruption in CNS cells, are other contributing factors (Brown et al., 2001; Marty et al., 2021). Certain viral proteins, like *Tat* or *gp120* from HIV, have neurotoxic effects and can lead to depression-like disorders (Lawson et al., 2011). The HIV nef protein in CNS microglia can cause hyperactivity, impulsivity, and impaired dopamine transmission, resulting in psychotic disorders (Acharjee et al., 2014). Other viral proteins with similar effects likely exist but are not yet identified. Indirect factors include coagulation disorders and endothelial damage, as seen in COVID-19 or *influenza A virus* (Fosse et al., 2021; Lamers and Haagmans, 2022). It is also possible that viruses increase the risk of mental illness only in individuals with other risk factors. For instance, psychotic disorders are more common in HIV patients with a history of drug abuse, suggesting that past drug use lowers the threshold for psychosis, with HIV acting as a trigger (Sewell et al., 1994). Stress and stigmatization associated with viruses like HIV and SARS-CoV-2 cannot be ignored as well. Indirect evidence of the virus-mental illness link includes the fact that antiviral drugs like HAART can alleviate mental disorder symptoms, and some psychotropic drugs, such as clozapine, chlorpromazine, or SSRIs, have antiviral effects (Munjal et al., 2017; Otręba et al., 2020; Fred et al., 2021; Kutkat et al., 2022; Rybakowski, 2022). Factors contributing to the development of mental disorders are visually represented in **Figure 1**.

Certain factors complicate the assessment of the relationship between viral infections and mental illnesses. For instance, mental disorders may induce abnormalities in the immune response, or conversely, individuals with abnormal immune responses may be more susceptible to developing mental disorders. Regardless of the causal direction, these immunological disorders predispose individuals to more infections and related biochemical, metabolic, and CNS changes. If such immunological impairments exist, they would complicate the detection of infections and their impact on mental health (Dickerson et al., 2019). Another factor distorting this assessment is post-intensive care syndrome (PICS), which can occur after intensive treatment or multiple hospitalizations and can mimic psychiatric complications, further complicating assessments (Rawal et al., 2017; Taquet et al., 2021a). Risky behaviors and poor hygiene among psychiatric patients can lead to more frequent viral infections and skew results

### 4.1 Final conclusions

#### 4.1.1 Influence of viral infections on mental disorders

• Viral infections may influence the development of mental disorders.

• It is not possible to assess the risk based solely on the cytokine profile during infection.

#### 4.1.2 Risk assessment

• The risk of mental disorders can only be preliminarily evaluated, including the potential occurrence of psychotic symptoms.

• Major cytokine disturbances may modulate the risk of mental disorders by sensitizing individuals to other risk factors, such as environmental or genetic factors.

#### 4.1.3 Contributing factors

• Specific viral mechanisms, the life cycle of certain viruses, the neurotoxic proteins they produce, molecular damage, and non-immunological changes like endothelial dysfunction and thromboembolic disorders may significantly contribute to the risk of mental disorders.

#### 4.1.4 Comprehensive assessment

• When assessing the risk of developing mental disorders in infected patients, it is imperative to:

o Consider the type of virus

o Scrutinize the mental complications most frequently associated with it.

o Evaluate the dominant cytokines to assess the risk of psychotic symptoms (particularly IL-8 and IL-10).

o Account for additional risk factors present in the patient, such as drug history, family history, and past mental illness.

#### 4.1.5 Future research

• Research should focus on specific viral mechanisms, not limited to inflammation-induced disorders.

• Studies on specific viral proteins and the identification of those with neurotoxic properties will be crucial.

• An in-depth analysis of immunological and inflammatory disorders, as well as changes in the human proteome and metabolome induced by viruses and in individuals suffering from mental disorders, may be the most useful approach to understanding the relationship between viruses and mental illnesses, as well as the etiology of mental disorders.

• It is important that these studies be conducted on large populations and have a prospective nature.

• Psychiatric monitoring of children whose mothers were infected with SARS-CoV-2 during pregnancy will help assess the risk of mental disorders associated with prenatal exposure to this virus, which is crucial given the widespread exposure.

#### 4.1.6 Clinical implications

• To assist clinicians, **Table 5** provides an overview of potential mental health complications related to viral infections in patients

**Table 5:** Mental health complications related to viral infections.

VIRUS	THE MOST COMMON DISORDER	CYTOKINE PROFILE
**Influenza**	SCZ, BD	SCZ, FEP, ASD – mild course SCZ, BD – severe course
**HSV**	SCZ	SCZ, BD
**CMV**	MDD/BD	SCZ, BD
**EBV**	MDD	SCZ
**BoDV**	MDD	Non-specific
**RV**	ASD	Impossible to evaluate/interferonopathy
**HIV**	MDD, FEP	SCZ
**SARS-CoV-2**	MDD, AD, SD	SCZ, BD, FEP - Non-specific

The table below shows which mental disorder is most often described as associated with specific viral infections versus the mental disorder that should occur judging by the similarity in the cytokine profile. AD, anxiety disorder; ASD, autism spectrum disorder; BD, bipolar disorder; BoDV, borna disease virus; CMV, cytomegalovirus; EBV, Ebstein-Barr virus; FEP, first episode psychosis; HIV, human immunodeficiency virus; HSV, herpes simplex virus; MDD, major depressive disorder; SARS-CoV-2, Severe Acute Respiratory Syndrome Coronavirus 2; SD, sleep disorder.

## 5 Limitations of the study

This study is a narrative review, and objective statistical methods were not used to create cytokine profiles. Non-English publications were excluded, which limits the information pool and may introduce bias. Additionally, the studies we relied on may have contained bias, which our review may inadvertently perpetuate. Some source studies were based on archival material, which may have deteriorated or lacked objective laboratory methods for detecting infections. Moreover, several studies were from the mid-20th century and may reflect the knowledge of that time, not supported by modern scientific and laboratory methods.
